# Lymphangiogenesis Is Required for Pancreatic Islet Inflammation and Diabetes

**DOI:** 10.1371/journal.pone.0028023

**Published:** 2011-11-23

**Authors:** Na Yin, Nan Zhang, Girdhari Lal, Jiangnan Xu, Minhong Yan, Yaozhong Ding, Jonathan S. Bromberg

**Affiliations:** 1 Center for Vascular and Inflammatory Diseases, University of Maryland, Baltimore, Maryland, United States of America; 2 Departments of Surgery and Microbiology and Immunology, University of Maryland, Baltimore, Maryland, United States of America; 3 Department of Surgery, Marshall University, Huntington, West Virginia, United States of America; 4 Department of Tumor Biology and Angiogenesis, Division of Research, Genentech Inc., South San Francisco, California, United States of America; La Jolla Institute of Allergy and Immunology, United States of America

## Abstract

Lymphangiogenesis is a common phenomenon observed during inflammation and engraftment of transplants, but its precise role in the immune response and underlying mechanisms of regulation remain poorly defined. Here we showed that in response to injury and autoimmunity, lymphangiogenesis occurred around islets and played a key role in the islet inflammation in mice. Vascular endothelial growth factors receptor 3 (VEGFR3) is specifically involved in lymphangiogenesis, and blockade of VEGFR3 potently inhibited lymphangiogenesis in both islets and the draining LN during multiple low-dose streptozotocin (MLDS) induced autoimmune insulitis, which resulted in less T cell infiltration, preservation of islets and prevention of the onset of diabetes. In addition to their well-known conduit function, lymphatic endothelial cells (LEC) also produced chemokines in response to inflammation. These LEC attracted two distinct CX3CR1^hi^ and LYVE-1^+^ macrophage subsets to the inflamed islets and CX3CR1^hi^ cells were influenced by LEC to differentiate into LYVE-1^+^ cells closely associated with lymphatic vessels. These observations indicate a linkage among lymphangiogenesis and myeloid cell inflammation during insulitis. Thus, inhibition of lymphangiogenesis holds potential for treating insulitis and autoimmune diabetes.

## Introduction

Lymphatics undergo growth and remodeling during many pathophysiological processes [1], [2], [3], [4], [5], and contribute to immunity during tumor growth and metastases [1], [2]. However, little is known about the mechanisms regulating lymphatics during inflammation, nor how lymphatics influence the progression of immune response. Islets had been considered to lack functional lymphatic vessels [6]. However, studies in NOD mice show that lymphatic vessels are adjacent to inflamed islets [7], [8] and a functional lymphatic network is also found in transplanted islets [9], suggesting peri- and intra-islet lymphatics might be involved in regulating islet inflammation. However, the significance of islet lymphangiogenesis and its contribution to islet inflammation remains elusive.

With the identification of relatively specific lymphatic markers such as VEGFR3, LYVE-1 [10], podoplanin [11], and Prox-1 [12], and the development of anti-lymphatic agents, the mechanisms of lymphatic function have started to be elucidated [13], [14]. VEGFR3 is present in all endothelia during early stages of development, and *Vegfr3* gene-targeted mice die at around E10.5 due to defective development of the cardiovascular system [2], [15]. The expression of VEGFR3 becomes restricted exclusively to LEC with the exception of corneal dendritic cells and some angiogenic blood vessels in tumors and healing wounds [15], [16]. Vascular endothelial growth factors VEGF-C and -D are the most potent inducers of lymphatic growth via VEGFR3 [13], [14], [17], [18]. VEGF-A, the primary blood angiogenic factor binding to VEGFR2, signals the major pathway to activate angiogenesis [13], [14]. VEGF-C also binds to VEGFR2, which is expressed predominantly on blood vessels, but also to a small extent on lymphatic vessels [18], [19]. A recent study showed that both VEGFR2 and VEGFR3 mediate VEGF-A induced inflammatory cutaneous lymphangiogenesis [20]. Lymphangiogenesis can be inhibited by VEGF-C/-D trap, neutralizing anti-VEGFR3 antibodies, or tyrosine kinase inhibitors, such as sunitinib, which inhibit VEGFR3 signaling [21].

Here, we explored the roles of lymphangiogenesis and lymphatic conduit function in islet inflammation. We showed that lymphangiogenesis occurred both in inflamed islets and the draining LNs, and prevention of diabetes was associated with inhibition of lymphangiogenesis. The production of potent lymphangiogenic and chemotactic molecules by LEC which attracted the myeloid cells linked inflammation and lymphangiogenesis. Inhibition of lymphangiogenesis decreased macrophage and T cell infiltration, preserved islet architecture and function, and prevented diabetes. These findings demonstrate important and novel communications between the myeloid and lymphatic systems to regulate adaptive immune responses.

## Materials and Methods

### Mice

BALB/c and C57BL/6 mice were from Jackson Laboratory (Bar Harbor, ME). CX3CR1^GFP/GFP^ on the C57BL/6 and BALB/c backgrounds were from Dr. Littman (Skirball Institute, New York, NY). CX3CR1^GFP/GFP^ C57BL/6 mice were crossed with C57BL/6 mice to produce CX3CR1^GFP/+^. All mice were housed in a pathogen-free animal facility and all efforts were made to minimize suffering. Mice were anesthetized with carbon dioxide. All experimental protocols involving mice were approved by the Institutional Animal Care and Utilization Committee of University of Maryland Medical Center (protocol # 0610003 and 0610004).

### Diabetes Induction and Agent Administration

Male BALB/c mice (8–10 weeks old) were given intraperitoneal injections of streptozotocin (STZ, Sigma-Aldrich, St. Louis, MO), at a dose of 40 mg/kg daily for 5 consecutive days. Animals were considered diabetic when blood glucose were >300 mg/dl for 2 consecutive days. FTY720 was from Dr. V. Brinkmann (Novartis Pharma, Basel, Switzerland). Rat anti-VEGFR3 mAb (mF4-31C1) was from Dr. B. Pytowsky (ImClone Systems, New York, NY) [22]. Sunitinib (sunitinib malate, SU-11248-L) was from Dr. James Christensen (Pfizer, Inc., Groton, CT). ALK1-Fc (ALK1 human IgG1) was previously described [23]. Control human-IgG1, rat IgG1 (HRPN) and rat anti-VEGFR2 mAb (DC101) were from Bio X Cell (West Lebanon, NH). The dose of treatment chosen for each was based on previous experience: FTY720 (1 mg/kg) and sunitinib (40 mg/kg) were administered by oral gavage (once daily) [24], [25], and PBS, anti-VEGFR3 (32 mg/kg) [22], [26], rat IgG1 (35 mg/kg), and anti-VEGFR2 mAb (35 mg/kg) [20] were administered by intraperitoneal injection (3 times per week), for 2 weeks starting from the first STZ injection. Activin receptor-like kinase 1 (ALK1)-Fc and human IgG1 (10 mg/kg) was administered by intraperitoneal injection twice weekly for 4 weeks starting from the first STZ injection [23].

### Immunofluorescent staining and quantitative image analysis

Hand-picked islets were incubated with anti-LYVE-1 and FITC-anti-CD31, followed by Cy5-conjugated goat anti-rabbit IgG, and then mounted with Vectashield (with DAPI) (Vector Laboratories, Inc., Berlingame, CA). Eight-10 µm frozen sections of LNs and pancreas were fixed by acetone. Cultured cells grown on chamber slide were fixed by 4% paraformaldehyde. After blocking with PBS/5% donkey serum, sections were incubated with primary antibodies, and followed by secondary antibodies in PBS/1% donkey serum, and sections were mounted with Vectashield. Purified rat anti-peripheral LN addressin (PNAd, MECA79), American hamster anti-CD3ε (145-2C11), rat anti-MECA32 and rat anti-CD31 (390) were from BD Biosciences-Pharmingen (San Jose, CA). Guinea pig anti-swan insulin was from Dako Cytomation, Inc. (Carpinteria, CA). Purified rabbit anti-LYVE-1 was from Fitzgerald Industries International, Inc. (Concord, MA). Purified rat anti-CD68 (FA-11) was from AbD Serotec, MorphoSys UK Ltd (Oxford, UK). Cy5 or Cy3-conjugated goat anti-rabbit IgG, DyLight488 or DyLight649-conjugated donkey anti-rabbit IgG, Cy3 or DyLight488-conjugated goat anti-hamster IgG, DyLight649-conjugated donkey anti-rat IgG, FITC-conjugated goat anti-rat IgM and Cy3-conjugated goat anti-guinea pig IgG were from Jackson ImmunoResearch Laboratories, Inc. (West Grove, PA). Images were acquired with a Leica DMRA2 fluorescent microscope (Leica Microsystems, Wetzlar GmbH, Germany), and Openlab or Volocity (Improvision, Inc., MA). Quantitative analysis was performed with Image J (NIH, Bethesda, MD) or Volocity. Two-five tissue sections from each pancreas or LN were randomly chosen. In each section of pancreas, all islet-centered areas (about 350–450 pixels at 200× magnification to the edge of islet) were selected and the specific positively staining areas were measured by Image J software. The areas within and around islets were manually separated according to DAPI and insulin staining. The areas covered by CD3 or insulin staining inside the islet boundaries were measured respectively, and expressed as a percentage of the whole islet area. The areas covered by LYVE-1 around the islet were measured as the density of lymphatic vessels and expressed as a percentage of the whole area outside of the islet. The positive areas for blood vessels (MECA32^+^) within or around the islet were measured separately, and the density was expressed as a percentage of the whole islet areas or the whole outside areas. CD68^+^LYVE-1^−^ cells and CD68^+^LYVE-1^+^ cells touching islets were manually counted with Volocity. The circumference of each islet was measured by Volocity. Macrophage numbers of per µm in each islet from each group were compared. LN lymphatic vessels and HEVs were measured as LYVE-1 or PANd positively staining areas by Image J, respectively; and expressed as a percentage of the whole LN area.

### Flow cytometry

Pancreata were digested with 8 mg/ml collagenase-D (Roche Diagnostics, Indianapolis, IN) with 10 mg/ml DNase I (Sigma) for 60 minutes at 37°C. Granulocytes, erythrocytes and dead cells were removed by Ficoll-Paque (GE Healthcare, Piscataway, NJ) density gradient centrifugation. Pancreatic cells were resuspended in PBS containing 1% BSA, 2 µg/ml FcR-blocking buffer (eBioscience Inc., San Diego, CA) and 1 mM EDTA, and stained with antibodies at 4°C. Fluorescent or biotin labeled antibodies against mouse podoplanin (8.1.1), CD31 (390), CD45 (30-F11), CD11b (M1/70), F4/80 (BM8), LYVE-1 (ALY7) and CD11c (N418) were from eBioscience. Fluorescent- or biotin-labeled anti-CD68 (FA-11) were from AbD Serotec. CD68 was stained extracellularly and subsequently intracellularly with the Cytofix/Cytoperm kit (BD) according to the manufacturer's protocol. Flow cytometric analyses were performed on an LSRII flow cytometer (BD) with FlowJo (Tree Star Inc.). Dead cells were excluded by DAPI (Invitrogen) staining.

### Pancreatic macrophage and LEC isolation

CX3CR1^hi^Gr1^med^LYVE-1^−^ and CX3CR1^lo^Gr1^med^LYVE-1^hi^ macrophages, and CD45^−^LYVE-1^+^ LEC were isolated from pancreatic single-cell suspensions from normal or MLDS treated CX3CR1^GFP/+^ mice by Aria II flow cytometer (BD). Sorted cells were spun onto the slides, and stained with Wright's stain (Sigma) after methanol fixation.

### Tube formation

Sorted CX3CR1^hi^ macrophages and lymphocytes (CD45^+^CD11b^−^Gr1^−^) were labeled with CFSE (Invitrogen), and LYVE-1^+^ macrophages were labeled with eFlour670 (eBioscience) before culture. Matrigel (BD) diluted with EBM-2 Endothelial Basal Medium (Lonza Inc., Walkerville, MD) (Matrigel∶EBM-2, 2∶1) was added to 8-well chamber slides (LAB-TEK, Naperville, IL) and allowed to gel at 37°C. For mixed cultures, 5×10^4^ CX3CR1^hi^ macrophages-CFSE, 5×10^4^ LYVE-1^+^ macrophages-eFlour670, 5×10^4^ lymphocytes-CFSE and/or 1×10^4^ LEC in Endothelial Cell Growth Medium-2 were then seeded onto the Matrigel and cultured for 5 days. For staining, 1×10^5^ CX3CR1^hi^ macrophages-CFSE with/without 1×10^4^ LEC were directly seeded onto *CC* ^2^ coated 8-well chamber slides. After 5 days, cells were stained for CD11b and LYVE-1.

### Quantitative real-time PCR (qRT-PCR)

The procedures for RNA isolation, cDNA synthetic and quantification by qRT-PCR were described previously [24]. qPCR used Oligo(dT) primers on a the LightCycler 2.0 (Roche) machine. Relative expression was calculated as 2^cycle threshold [Ct] control – Ct gene^ using cyclophilin A as an endogenous control. Primer sequences for CCR8, forward 5′-TGACCGACTACTACCCTGATTTCTT-3′ and reverse 5′- GCTGCCCCTGAGGAGGAA-3′; CCL2 forward 5′-GATCCCAATGAGTAGGCTGG-3′ and reverse 5′-CGGGTCAACTTCACATTCAAAG-3′; CCL3 forward 5′-CCAAGTCTTCTCAGCCAT-3′ and reverse 5′-TCCGGCTGTAGGAGAAGCAG-3′; CCL4 forward 5′-CCACTTCCTGCTGTTTCTCT-3′ and reverse 5′-CACAGATCTGTCTGCCTCTT-3′; CCL5, forward 5′-CAAGTGCTCCAATCTTGCAGTC-3′ and reverse 5′-TTCTCTGGGTTGGCACACAC-3′; CCL8 forward 5′-ACAATATCCAGTGCCCCATG -3′ and reverse 5′-GATGAGAAAACACGCAGCC-3′; CXCL10 forward 5′-CCTGCTGGGTCTGAGTGGGA-3′ and reverse 5′-GATAGGCTCGCAGGGATGAT-3′; CX3CL1 forward 5′-ACAAGATGACCTCACGAATCC-3′ and reverse 5′-TCCACCCGCTTCTCAAAC-3′, CCL21 forward 5′-GGGAACCTCTAAGTCTGGAAAG-3′ and reverse 5′-GGCTCCTGAGTCTGTTTTCTAG-3′. Other primers were from previous publications [24], [27].

### Statistical Analysis

Each histological parameter was measured in a blinded fashion. The differences were assessed using unpaired Student's t test and expressed as the mean ± standard deviation (SD). A Value of p<0.05 was taken to be statistically significant. Diabetic incidence curves were constructed with Kaplan-Meier estimates and analyzed by the generalized Wilcoxon's test.

## Results

### MLDS-induced islet inflammation causes lymphangiogenesis

**Figure 1:**
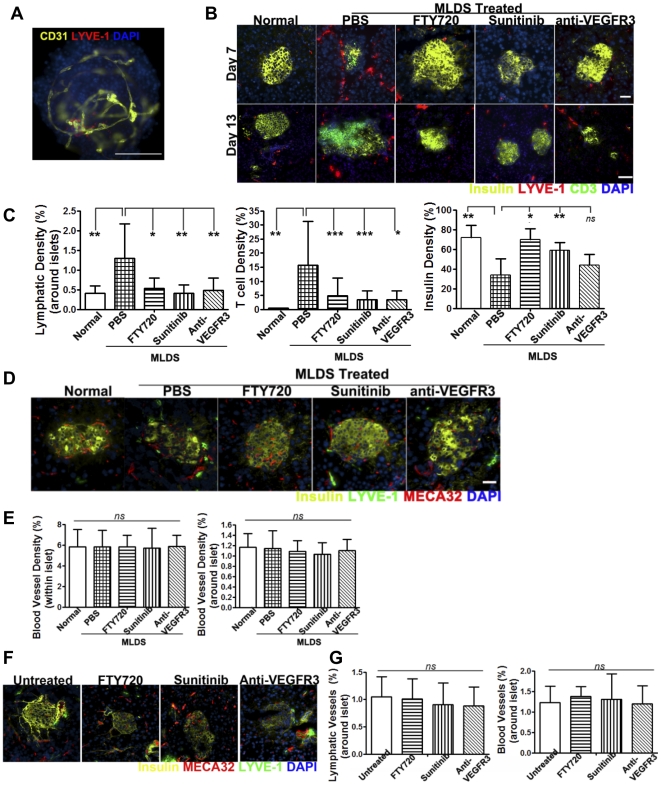
MLDS-induced islet inflammation causes lymphangiogenesis. MLDS treated BALB/c mice received FTY720, sunitinib, or anti-VEGFR3 mAb starting from the first STZ injection. (A) Whole mount immunohistochemistry of isolated normal islets of BALB/c mice. Blood vessels: CD31; lymphatic vessels: LYVE-1. Scale bar: 200 pixels. 200× magnification. (B) Immunofluorescent analysis of beta-cells (insulin), T cells (CD3) and lymphatic vessels (LYVE-1) in pancreas 7 days or 13 days after initiation of MLDS treatment. Scale bars: 32 µm. (D) Immunofluorescent analysis of beta-cells (insulin), blood vessels (MECA32) and lymphatic vessels (LYVE-1) 7 days after initiation of MLDS treatment. Scale bars: 32 µm. (C) and (E) Quantitative analysis of insulin, CD3, LYVE-1 and MECA32 staining of pancreas 7 days after initiation of MLDS treatment. 12–15 islets for insulin and CD3, 12–15 areas around islets for LYVE-1, and 14–21 islets or areas around islets for MECA32; 2 slides/mouse; 2–4 mice/group. * P≤0.05, ** P≤0.01, *** P≤0.001; *ns*, not significantly. (F) and (G) Normal BALB/c mice received indicated treatment for 7 days. (F) Immunofluorescent analysis of beta-cells (insulin), lymphatic vessels (LYVE-1) and blood vessels (MECA32). 200× magnification. (G) Quantitative analysis of LYVE-1 and MECA32 staining of pancreas. 18–21 areas around islets; 2 slides/mouse; 2–4 mice/group. P>0.1 *vs* untreated mice. Mean ± SD. 200× magnification.

To examine the vascular and lymphatic networks, isolated islets were stained for the lymphatic and blood endothelial markers LYVE-1 and CD31, respectively. A CD31^+^ blood vessel network was contained within normal naive islets, while LYVE-1^+^ lymphatic vessels surrounding some of islets penetrated only into the hilum (Figure 1A). To investigate the role of islet lymphatic vessels in diabetes, BALB/c mice were rendered diabetic by MLDS, which induces islet inflammation and T cell dependent autoimmunity [28]. Mice were treated with anti-VEGFR3 mAb, sunitinib, or FTY720 starting on the first day of STZ administration. Pancreata were examined 7–13 days after treatment by immunofluorescent staining for insulin, LYVE-1 and CD3. MLDS caused T cell infiltration, islet destruction and loss. LYVE-1^+^ lymphatic vessels around the islets increased after MLDS, demonstrating inflammation caused lymphangiogenesis. FTY720, an S1P1 modulator [24] which has been shown to prevent diabetes in NOD mice and islet rejection [29], [30], prevented loss of insulin^+^ cells, T cell infiltration and lymphangiogenesis, demonstrating that lymphangiogenesis is involved in islet inflammation. Both anti-VEGFR3 mAb, which specifically targets LEC growth receptors, and sunitinib, which inhibits the kinase activity of VEGFR3, inhibited islet lymphangiogenesis, reduced or prevented loss of insulin^+^ cells and reduced T cell infiltration (Figure 1B–1C). MLDS did not induce blood venule angiogenesis within or around the islets, and FTY720, sunitinib and anti-VEGFR3 mAb did not influence blood vessel density (Figure 1D–1E). Thus, blood vessel angiogenesis was not induced and angiogenesis was less important for MLDS-induced islet inflammation. Since sunitinib can also inhibit kinases involved in blood vascular angiogenesis, this showed that sunitinib prevented islet inflammation mainly through inhibiting lymphangiogenesis. The effects of these inhibitors on existing lymphatic and blood vessels were examined in non-inflamed tissues. Neither lymphatic nor blood vessel density around the islets were influenced by FTY720, sunitinib or anti-VEGFR3 in normal mice, demonstrating that these reagents inhibited inflammation-induced lymphangiogenesis, but did not affect existing lymphatic and blood vessels (Figure 1F–1G). Overall, prevention of beta-cell loss correlated with inhibition of lymphangiogenesis rather than a change in angiogenesis in islets.

**Figure 2:**
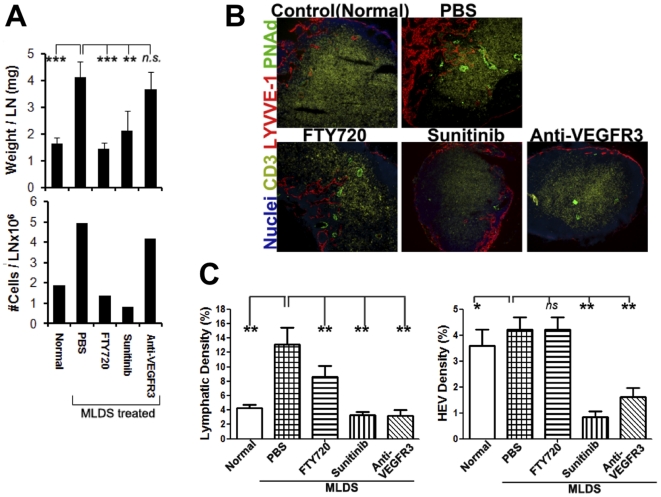
Inhibition of lymphatic function alters draining LN size and prevents MLDS induced LN lymphangiogenesis. MLDS treated BALB/c mice received FTY720, sunitinib, or anti-VEGFR3 mAb starting from the first STZ injection. Mice were euthanized 7 days after initial MLDS treatment. (A) Weight (n = 5 mice/group) and cell number (pooled 2 LNs) of draining pancreatic LNs. (B) Immunofluorescent analysis of T cells (CD3), lymphatic vessels (LYVE-1) and HEVs (PNAd) in pancreatic LNs 7 days after initiation of MLDS treatment. 100× magnification. (C) Quantitative analysis of LYVE-1 and PNAd staining in draining pancreatic LNs. 2 slides/mouse, 2–4 mice/group. Mean ± SD. * P≤0.01, **, P≤0.001.

LNs play key roles in inflammation and immune responses [31]. As expected the pancreatic draining LNs of treated mice enlarged, and sunitinib and FTY720 inhibited this LN response (Figure 2A). Draining LNs were evaluated for lymphangiogenesis and high endothelial venule (HEV) angiogenesis by staining for LYVE-1 and PNAd, respectively. MLDS stimulated LN lymphangiogenesis (Figure 2B–2C), but did not cause HEV vascular angiogenesis; inhibition of inflammation with the immunosuppressant FTY720 inhibited LN lymphangiogeneiss but not HEV angiogenesis, suggesting that LN lymphangiogeneis was more related to islet inflammation than HEV angiogenesis. Both sunitinib and anti-VEGFR3 mAb prevented LN lymphangiogenesis. Sunitinib and anti-VEGFR3 mAb also both markedly reduced the extent of PNAd^+^ HEVs, likely reflecting the utilization of VEGFR3 by blood vascular endothelial cells [17], [18]. LN responses are the result of a number of distinct factors and LN responses were likely reduced both by inhibiting upstream islet lymphangiogenesis and conduit function, and downstream intranodal lymphangiogenesis. Together these results demonstrated a tight association between tissue and LN lymphatic function and lymphangiogenesis.

### Inhibition of lymphangiogenesis prevents MLDS-induced diabetes

**Figure 3:**
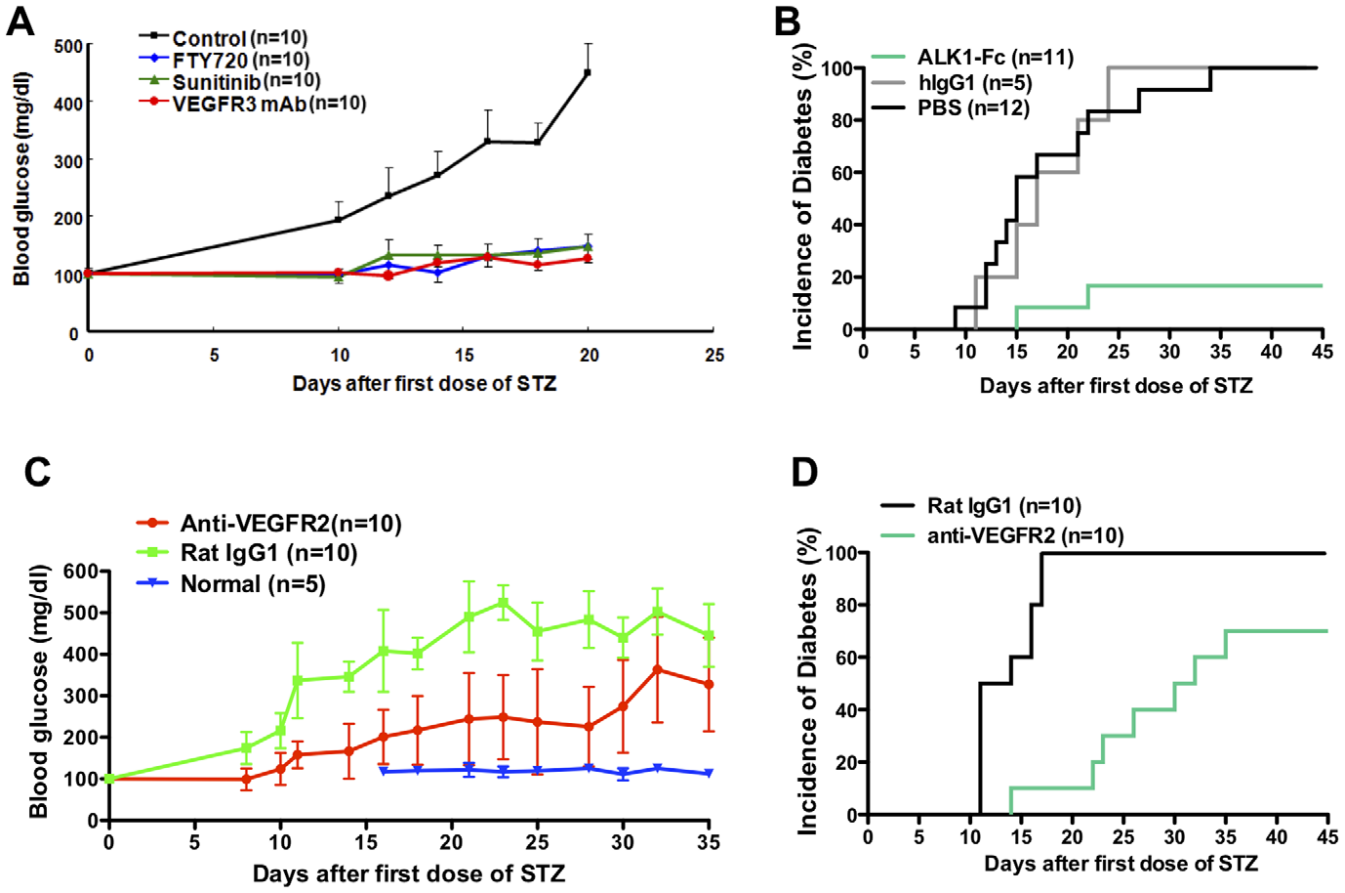
Inhibition of lymphatic function prevents diabetes by MLDS treatment. (A) MLDS treated BALB/c mice received FTY720, sunitinib, or anti-VEGFR3 mAb starting from the first STZ injection for 2 weeks. Blood glucose profiles, compared to control of MLDS treated mice receiving the indicated treatments. P-values for all groups compared to control, <0.001. (B) Incidence of diabetes. MLDS treated mice received ALK1-Fc, control human IgG1 or PBS starting from the first STZ injection for 4 weeks. (C and D) MLDS treated BALB/c mice received anti-VEGFR2 mAb or control rat IgG1 starting from the first STZ injection for 2 weeks. (C) Blood glucose profiles, p-values all <0.05, anti-VEGFR2 compared to rat IgG1 or normal. (D) Incidence of diabetes, p = 0.0002.

To define the role of lymphangiogenesis in diabetes, we investigated the roles of distinct lymphangiogenic and angiogenic inhibitors in MLDS induced diabetes. As shown in Figure 3A, blood glucose levels increased by 10 days after STZ initiation. Similar to FTY720, both anti-VEGFR3 mAbs and sunitinib completely prevented hyperglycemia (Figure 3A). ALK1 is a member of the transforming growth factor-β type I family of receptors and is primarily expressed in the developing vascular system, blockade of ALK1 signaling results in defective lymphatic vascular development and ALK1-Fc blocks its function [23]. ALK1-Fc also prevented diabetes in over 80% of mice (Figure 3B). Thus, inhibition of lymphangiogenesis or lymphatic development prevented MLDS induced insulitis and autoimmune diabetes. In contrast, anti-VEGFR2 mAb delayed the day of onset and only decreased but did not prevent the incidence of hyperglycemia (Figure 3C), preventing MLDS induced diabetes in only 30% of treated mice (Figure 3D), indicating that blockade of VEGFR2 prevented insulitis to a much lesser extent compared to blockade of VEGFR3.

### Lymphatic endothelial cells produce chemokines and VEGFs

**Figure 4:**
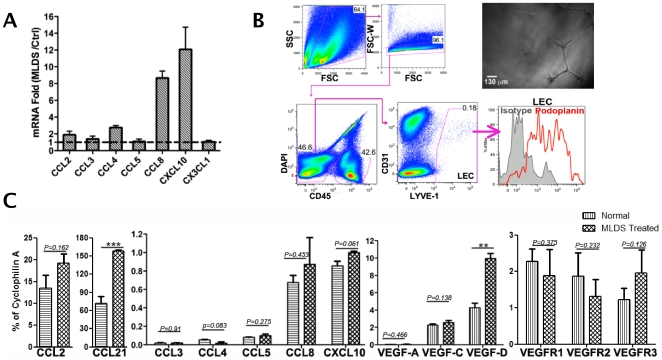
Expression of VEGFs and chemokines by pancreas and lymphatic endothelial cells. (A) mRNA expression of chemokines in pancreas of MLDS treated BALB/c mice determined by qRT-PCR before treatment and on day 7. Values expressed as fold change. 3 mice/group, data representative of 2 separate experiments. (B) Flow sorting gates. LEC gated on FSC-A *vs* SSC-A, FSC-A *vs* FSC-W, CD45^+^, DAPI^−^, CD31^+/−^ and LYVE-1^+^. Right upper panel, sorted LEC (LYVE-1^+^CD31^+/−^CD45^−^) cultured on Matrigel for 5 days. 100× magnification. (C) mRNA expression profile of chemokines and VEGFs in sorted pancreatic LEC. CD45^−^CD31^+/−^LYVE-1^+^ cells sorted from normal or MLDS treated CX3CR1^GFP/+^ (day 3 after initial treatment), 6–7 mice/group. Data representative of 2 separate experiments. PCR performed in triplicate. * P≤0.05, ** P≤0.01, *** P≤0.001. All data are represented as mean ± SD.

To dissect the events linking inflammation and lymphangiogenesis, whole pancreas was examined for expression of inflammatory mediators. Total RNA from pancreata before and on day 7 after MLDS was assayed. Levels of CCL8 and CXCL10 were increased by 8- to 12-fold after MLDS treatment, and CCL2 and CCL4 were increased by 2- to 3-fold (Figure 4A). Importantly, CCL2, 4, 8 and CXCL10 recruit monocytes/macrophages and T cells to sites of tissue injury and inflammation [32], suggesting MLDS-induced tissue damage directly facilitated monocyte and T cell infiltration. We previously demonstrated by gene-array analysis that LEC line SVEC4-10 expressed various chemokines at baseline and in response to inflammatory stimuli, suggesting that LEC may be a source of chemokine in MLDS [24]. Therefore, we examined the expression of chemokines and VEGFs in freshly isolated pancreatic LECs, which were obtained before and on day 3 after MLDS. Pancreatic LEC expressed podoplanin (Figure 4B) and VEGFRs (Figure 4C) and formed tube-like structures in culture (Figure 4B), demonstrating their LEC phenotype. Expression of mRNA for CCL2 and CCL21 was observed, and after MLDS treatment CCL21 increased significantly and CCL2 increased less so. The expression of other chemokines was also observed. LEC expressed all three VEGFs. After MLDS, VEGF-D expression significantly increased (Figure 4C). These data supported the notion that LEC responded to initial inflammatory signals by producing chemokines that recruited additional inflammatory cells and by producing VEGFs that promoted lymphangiogenesis, thus contributing to islet inflammation in a paracrine manner.

### LEC attract two macrophage subsets which contribute to lymphangiogenesis and islet inflammation

**Figure 5:**
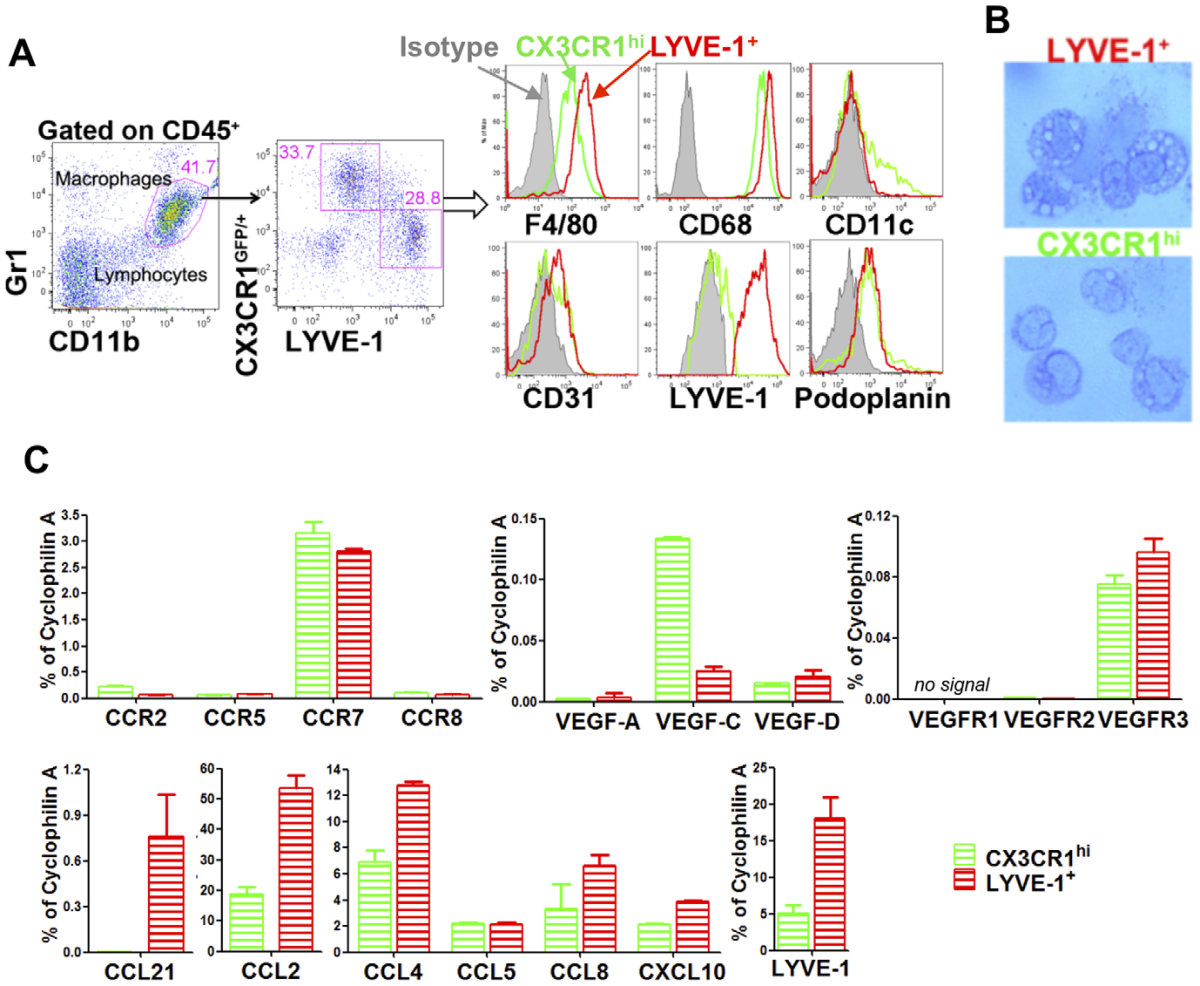
Phenotype of pancreatic macrophages. (A) Identification of macrophage subsets in pancreas of CX3CR1^GFP/+^ C56BL/6 mice. Pancreatic single cell suspensions were gated on FSC-A *vs* FSC-W and CD45^+^. Histograms show receptor expression profile of CX3CR1^hi^LYVE-1^−^ (green line) and CX3CR1^lo^LYVE-1^+^ (red line) macrophages. (B) Sorted CX3CR1^lo^LYVE-1^+^ and CX3CR1^hi^LYVE-1^−^ macrophages spun onto glass slides and stained with Wright's stain. 1000× magnification. (C) mRNA expression profile of chemokine receptors, chemokines and VEGFs in pancreatic macrophage subsets. mRNA levels examined by qRT-PCR in duplicate or triplicate. 6–7 mice/group, data representative of 2–4 separate experiments.

Macrophages have been shown to play a role during the development of diabetes [33], [34], [35]. Recent reports also suggest that CD11b^+^Gr1^+^ macrophages contribute to lymphangiogenesis in trachea [36], cornea [37], [38], skin [39] and tumors [40]. We analyzed macrophage phenotype in pancreas in normal mice. As shown in Figure 5A, the majority of pancreatic CD11b^+^ cells were Gr1^med^. The Gr1^med^CD11b^+^ population was further divided by expression of the fractalkine receptor (CX3CR1) and LYVE-1, defining CX3CR1^hi^ (CX3CR1^hi^LYVE-1^−^) and LYVE-1^+^ (CX3CR1^lo^LYVE-1^+^) subsets. Both subsets expressed the macrophage markers F4/80 and CD68. Wright's stain showed that both subsets were mononuclear with a large nucleus and vacuolar cytoplasm (Figure 5B), indicative of macrophages. Ten-20% of the CX3CR1^hi^ subset also expressed low levels of CD11c, suggesting subset heterogeneity. The LYVE-1^+^ subset expressed higher levels of F4/80 and CD68, and had more and larger vacuolar cytoplasm. The two subsets expressed similar levels of the LEC marker podoplanin and the endothelial marker CD31. Chemokine receptor expression showed that two subsets expressed comparable levels of CCR2, CCR5, CCR7 and CCR8 (Figure 5C). Since LEC expressed the CCR7 ligand CCL21, which was up-regulated during islet inflammation, this indicated that LEC had the potential to attract macrophages. The CX3CR1^hi^ subset expressed higher VEGF-C. The LYVE-1^+^ subset, but not the CX3CR1^hi^ subset, expressed CCL21 and also expressed higher levels of CCL2 and CCL4. Both subsets expressed the VEGFR3 but not the VEGFR1 or VEGFR2 blood endothelial markers. These results suggested that the pancreas contained at least two phenotypically distinct macrophage populations, and suggested the CX3CR1^hi^ subset could sustain lymphangiogenesis through VEGF-C, while the LYVE-1^+^ subset resembled LEC in their LYVE-1^+^ phenotype and by expressing multiple inflammatory chemokines to attract additional myeloid cells.

**Figure 6:**
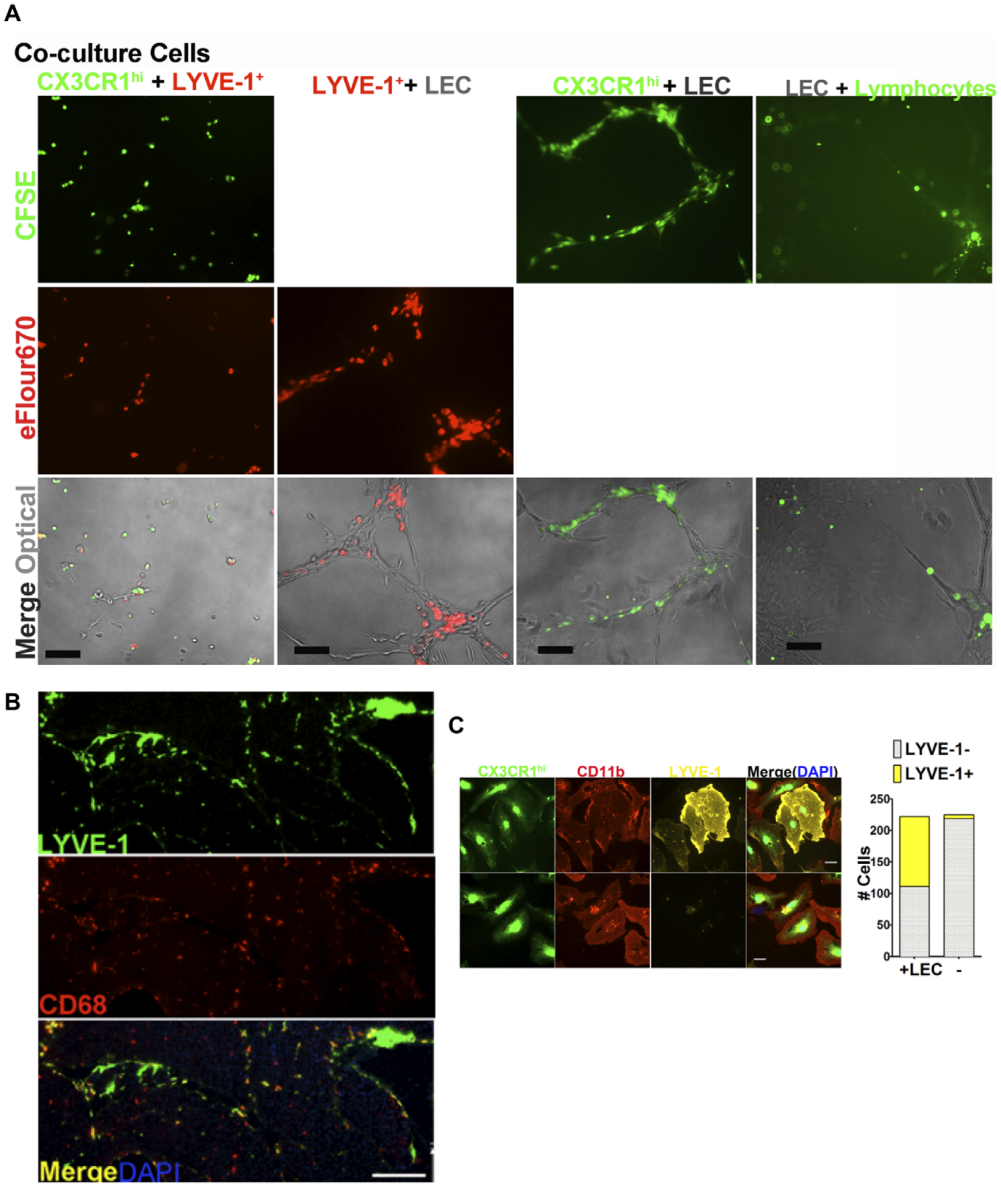
Interaction of LEC and pancreatic macrophages. (A) CX3CR1^hi^ macrophages, LYVE-1^+^ macrophages, CD11b^−^-lymphocytes and LEC were sorted from pancreatic single cell suspensions of CX3CR1^GFP/+^ mice. Co-cultured CX3CR1^hi^ macrophages-CFSE, LYVE-1^+^ macrophages-eFlour670, or lymphocytes-CFSE with/without LEC on Matrigel for 5 days. Scale bars: 60 µm. 100× magnification. (B) Immunofluorescent staining of CD68 and LYVE-1 in pancreas from C57BL/6 mice. Scale bars: 160 µm. 50× magnification. (C) Sorted CX3CR1^hi^ macrophages labeled with CFSE and co-cultured with LEC (left upper panel) or without LEC (left lower panel) for 5 days, and stained for CD11b (red) and LYVE-1 (yellow). Scale bars: 10 µm. 630× magnification. Right panel, quantitative analysis, total cells from 5 fields (1344×1024 pixels) were counted. All data representative of 2 to 4 separate experiments.

**Figure 7:**
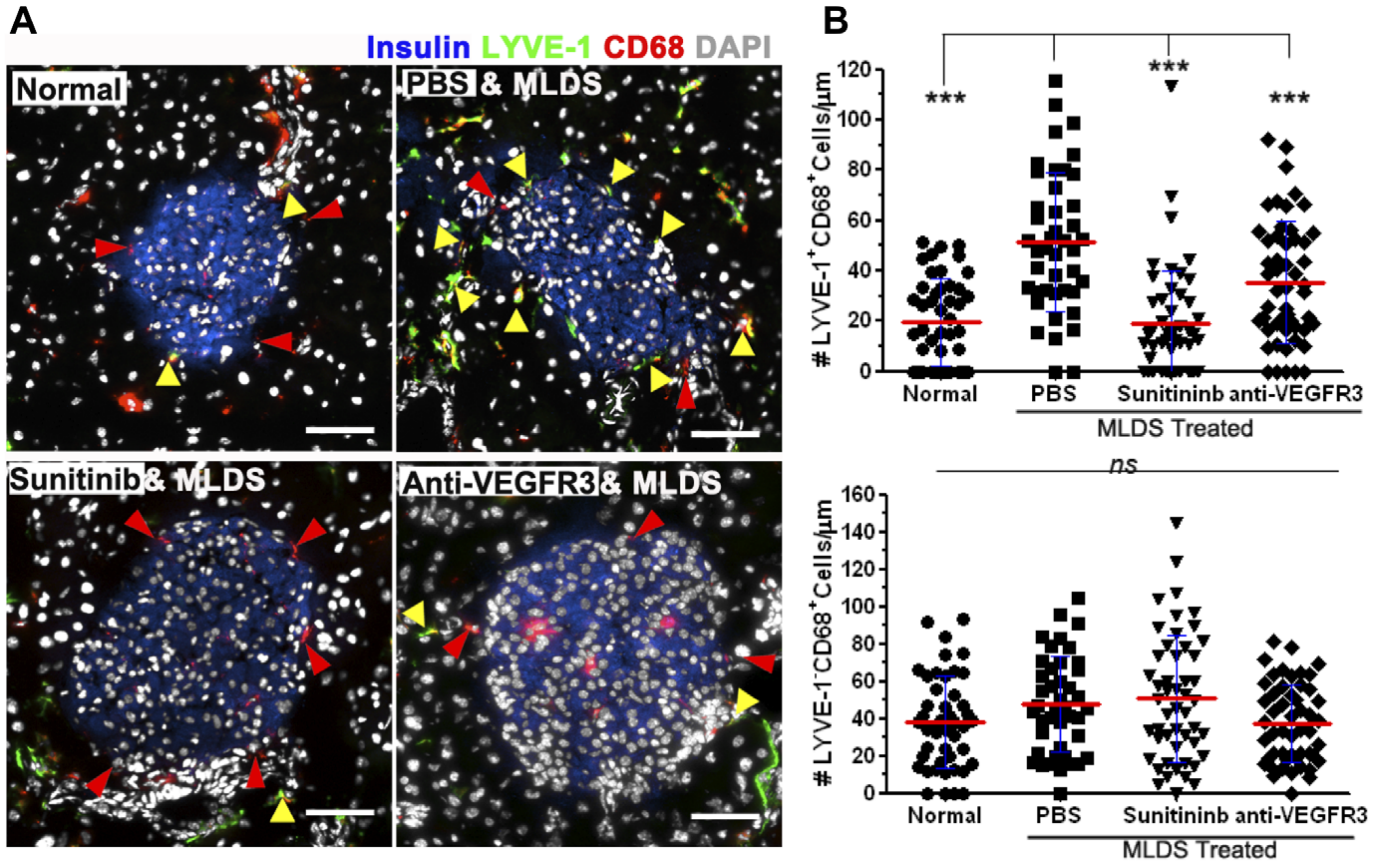
Macrophages infiltrate into inflamed islets. MLDS treated BALB/c mice received sunitinib, or anti-VEGFR3 mAb starting from the first STZ injection 3 days. (A) Immunofluorescent analysis of CD68^+^LYVE-1^+^ (yellow arrows) and CD68^+^LYVE-1^−^ (red arrows) macrophage subsets migrating near islets. 200× magnification. Scale bars: 30 µm. (B) Quantitative analysis of CD68^+^LYVE-1^+^ and CD68^+^LYVE-1^−^ cells surrounding islets. Each symbol represents one islet. 43–52 islets/group, 4–5 slides/mice, 3 mice/group. *** P≤0.001. Mean ± SD.

LEC cultured on Matrigel formed cord-like structures in vitro (Figure 4B), whereas macrophages distributed evenly in the culture in the absence of LEC (Figure 6A). When macrophages and LEC were co-cultured, both subsets became elongated, and lined up with and/or integrated into the cord-like structures of the LEC (Figure 6A); In contrast, lymphocytes remained rounded and were mostly found outside the LEC cords (Figure 6A). This indicated that LEC attracted and interacted with both macrophage subsets. Immunofluorescent staining of tissues (Figure 6B) showed that LYVE-1^+^CD68^+^ cells were lining or incorporated into lymphatic vessels in the pancreas. Isolated CFSE labeled CX3CR1^hi^ macrophages were cultured with pancreatic LEC for 5 days. Staining for LYVE-1 showed that 50% of CX3CR1^hi^CD11b^+^LYVE-1^−^ macrophages became LYVE-1^+^ after co-culture with LEC, but this change was not observed in the absence of LEC (Figure 6C). Thus, the CX3CR1^hi^ subset differentiated into the LYVE-1^+^ subset in a process dependent on LEC, while tightly associating with or integrating into LEC cord structures in vitro and in vivo. After MLDS induced inflammation, the LYVE-1^+^ macrophages surrounding islets significantly increased (Figure 7A–7B), suggesting LYVE-1^+^ macrophages migrated to the inflamed islets. Both sunitinib and anti-VEGFR3 inhibited LYVE-1^+^ macrophage accumulation around islets (Figure 7A–7B), suggesting that blockade of lymphangiogenesis reduced inflammation and inhibited recruitment of LYVE-1^+^ macrophages. Together, these data showed that LEC had the ability to recruit macrophages and influence their differentiation, while macrophages had the potential to sustain lymphangiogenesis and inflammation through the production of VEGFs and chemokines.

## Discussion

Islet inflammation occurs during type 1 diabetes and islet rejection, but the underlying mechanisms are not fully understood. In this study, we found that lymphangiogenesis played a pivotal role in MLDS induced islet inflammation and autoimmune insulitis. Blockade of lymphangiogenesis by anti-VEGFR3 as well as sunitinib inhibited insulitis, preserved islet beta-cells and prevented MLDS induced diabetes.

In contrast, other reports show blockade of VEGFR3 promotes the inflammatory process in chronic arthritis [41] and cutaneous inflammation in keratin 14-VEGF-A transgenic mouse models [20], [41], and specific activation of lymphatic vessels by overexpression of VEGF-C or VEGF-D, or by injection of VEGF-C attenuates inflammatory edema in both chronic and acute cutaneous inflammation models [20], [42]. It should be noted that there are abundant cutaneous lymphatic vessels present in the steady-state, and these reports [20], [42] demonstrated lymphatic vessel drainage and lymph flow are impaired during inflammation. Increased delivery of VEGF-C to the skin restores lymphatic function and improves edema resolution [20], [42]. Unlike skin, islets are largely devoid of lymphatic vessels in the steady-state. We observed that lymphangiogenesis was associated with T cell and macrophage infiltration during islet inflammation and blockade of VEGFR3 reduced inflammatory cell infiltration. Thus, in our model lymphangiogenesis contributed to the influx of an inflammatory infiltrate, while in the other models lymphangiogenesis overcame the dysfunction of lymphatics and contributed to the export of inflammatory cells and the resolution of inflammation. Our findings in this acute autoimmune inflammation model are in agreement with results of a previous study using an islet allogeneic transplantation model [43] and a corneal transplantation model [38], which showed inhibition of lymphangiogenesis prevented immune-mediated graft rejection. Lymphangiogenesis in kidney transplant is also associated with inflammatory lymphocytic infiltrates and transplant rejection [44]. Numerous CCR7^+^ cells are observed within the transplant kidney nodular and seem to be attracted by CCL21 released by LEC [44]. We also found that chemokines and VEGFs were up-regulated in LEC during islet inflammation. CCL21 expression in LEC is associated with activation of LEC via VEGFR3 signaling in cardiac allografts [45]. LPS-TLR4 signaling in LEC results in the production of various chemokines for chemotaxis of macrophages [46]. Besides CCL21, other mononuclear and T cell chemotactic factors were also up-regulated in LEC and the SVEC4-10 cell line (data not shown) in response to inflammatory stimuli. Thus lymphangiogenesis and activation of LEC have the potential to enhance leukocyte homing to the lymphatic vessels by affecting the chemotactic gradients [47], and enhance the initiation and maintenance of alloimmune and autoimmune responses in transplantation and MLDS-induced diabetes.

It has been reported that macrophages are the first cells that appear within the islets of NOD mice [34] and are required for the development and activation of the cytotoxic T cells that cause beta-cell destruction [35]. To our knowledge, this is the first characterization of pancreatic macrophage subsets in the pancreas. Our results showed that LEC attracted these macrophages and influenced the CX3CR1^hi^ subset differentiation into the LYVE-1^+^ subset. The two subsets displayed different phenotypes; the former produced higher VEGF-C and the latter displayed a lymphatic endothelial phenotype. Recent studies in transplantation, wound healing and tumor models show that macrophages are involved in lymphangiogenesis by secreting VEGFs to stimulate lymphatic vessel sprouting, and by differentiating into LEC and incorporating into lymphatic vessels [27], [39], [48], [49]. Thus recruited macrophages may enhance the lymphangiogenesis. Monocyte/macrophage infiltration also happens during cutaneous inflammation, and inhibition of VEGFR3 signaling decreases macrophage infiltration. Thus, macrophage infiltration seems associated with inflammation in the cutaneous inflammation model [20], [42]. Although the total number of macrophages in the whole pancreas did not change (data not shown), the number of LYVE-1^+^ macrophages significantly increased around inflamed islets and inhibition of lymphangiogenesis prevented LYVE-1^+^ macrophage infiltration. Thus newly formed lymphatics and activated LEC seemed able to induce relocation of macrophages within the pancreas to the inflamed islet. We propose a model where, under inflammatory stimuli, tissue LEC secrete chemokines and cytokines that recruit macrophages and influence their function.

Blood vessel angiogenesis caused by MLDS-induced islet inflammation was not observed until day 7, when macrophage and T cell infiltration, and lymphangiogenesis had already appeared. This implied that angiogenesis was not the main event that initiated islet immune responses. Blockade of VEGFR2, whose signaling is the major pathway that activates angiogenesis [13], [14], [15], delayed the progression of insulitis and partially prevented the onset of MLDS-induced diabetes. VEGFR2 has been implicated in lymphangiogenesis, as it has been reported to contribute to lymphangiogenesis during inflammation [18], [19], [20]. Thus, anti-VEGFR2 treatment might affect lymphangiogenesis but not to the same degree as anti-VEGFR3. VEGFR2 signals also regulate vascular permeability and adhesion molecules, and might further regulate immune response [50], [51].

The expression of VEGFR3 is restricted to LEC with few exceptions [13], [14], [16], [52]. Although one report showed VEGFR3 transcripts were detected in some human T cell lines [53], whether VEGFR3 expressed on surface of T cells remains to be proven and to our knowledge direct effects of VEGFR3 signaling on the function of leukocytes has not been reported. VEGFR3 expression was detected in these macrophages at the mRNA level, so it is possible that anti-VEGFR3 treatment directly altered macrophage recruitment and this needs further investigation. In this study, in addition to anti-VEGFR3 mAb, sunitinib was also used to interfere with lymphatic functions. Sunitinib is a potent anti-angiogenic and anti-lymphangiogenic kinase inhibitor that targets multiple tyrosine kinase receptors, including VEGFR, PDGFR, c-kit, and FLT3 [21], [25], [54], and is clinically approved for treatment of malignancies [54]. Recent reports use tyrosine kinase inhibitors for prevention of diabetes and diabetic complications [55], [56]. Louvet et al. [55] showed that sunitinib reversed new onset diabetes in NOD. They suggested that sunitinib inhibited PDGFR, although this specificity was not proven. So it also possible that sunitinib altered T cell recruitment or activation during islet inflammation. FTY720 is an agonist and antagonist for the S1P receptors [24], [57]. It has been reported that S1P induces lymphangiogenesis by stimulating the migration and differentiation of LEC via an S1P_1_/G_i_/PLC/Ca^2+^ signaling pathway [58]. Thus, FTY720 may have acted as an S1PR antagonist to inhibit LEC.

In summary, the results here suggest interplay between LEC and macrophages, with the former producing chemokines that attract the latter, which in turn express more chemokines and VEGFs to amplify lymphangiogenesis. Our study demonstrates that lymphangiogenesis plays a crucial role in insulitis suggesting that modulating islet lymphangiogenesis may serve important therapeutic effects in diabetes.

## References

[1] Sundar SS, Ganesan TS (2007). Role of lymphangiogenesis in cancer.. J Clin Oncol.

[2] Alitalo K, Tammela T, Petrova TV (2005). Lymphangiogenesis in development and human disease.. Nature.

[3] Angeli V, Ginhoux F, Llodra J, Quemeneur L, Frenette PS (2006). B cell-driven lymphangiogenesis in inflamed lymph nodes enhances dendritic cell mobilization.. Immunity.

[4] El-Chemaly S, Levine SJ, Moss J (2008). Lymphatics in lung disease.. Ann N Y Acad Sci.

[5] Oliver G, Alitalo K (2005). The lymphatic vasculature: recent progress and paradigms.. Annu Rev Cell Dev Biol.

[6] Regoli M, Bertelli E, Orazioli D, Fonzi L, Bastianini A (2001). Pancreatic lymphatic system in rodents.. Anat Rec.

[7] Qu P, Ji RC, Kato S (2003). Histochemical analysis of lymphatic endothelial cells in the pancreas of non-obese diabetic mice.. J Anat.

[8] Mounzer RH, Svendsen OS, Baluk P, Bergman CM, Padera TP (2010). Lymphotoxin-alpha contributes to lymphangiogenesis.. Blood.

[9] Kallskog O, Kampf C, Andersson A, Carlsson PO, Hansell P (2006). Lymphatic vessels in pancreatic islets implanted under the renal capsule of rats.. Am J Transplant.

[10] Banerji S, Ni J, Wang SX, Clasper S, Su J (1999). LYVE-1, a new homologue of the CD44 glycoprotein, is a lymph-specific receptor for hyaluronan.. J Cell Biol.

[11] Breiteneder-Geleff S, Matsui K, Soleiman A, Meraner P, Poczewski H (1997). Podoplanin, novel 43-kd membrane protein of glomerular epithelial cells, is down-regulated in puromycin nephrosis.. Am J Pathol.

[12] Wigle JT, Harvey N, Detmar M, Lagutina I, Grosveld G (2002). An essential role for Prox1 in the induction of the lymphatic endothelial cell phenotype.. EMBO J.

[13] Karpanen T, Alitalo K (2008). Molecular biology and pathology of lymphangiogenesis.. Annu Rev Pathol.

[14] Pepper MS, Skobe M (2003). Lymphatic endothelium: morphological, molecular and functional properties.. J Cell Biol.

[15] Tammela T, Alitalo K (2010). Lymphangiogenesis: Molecular mechanisms and future promise.. Cell.

[16] Hamrah P, Chen L, Zhang Q, Dana MR (2003). Novel expression of vascular endothelial growth factor receptor (VEGFR)-3 and VEGF-C on corneal dendritic cells.. Am J Pathol.

[17] Achen MG, Jeltsch M, Kukk E, Makinen T, Vitali A (1998). Vascular endothelial growth factor D (VEGF-D) is a ligand for the tyrosine kinases VEGF receptor 2 (Flk1) and VEGF receptor 3 (Flt4).. Proc Natl Acad Sci U S A.

[18] Joukov V, Pajusola K, Kaipainen A, Chilov D, Lahtinen I (1996). A novel vascular endothelial growth factor, VEGF-C, is a ligand for the Flt4 (VEGFR-3) and KDR (VEGFR-2) receptor tyrosine kinases.. EMBO J.

[19] Wirzenius M, Tammela T, Uutela M, He Y, Odorisio T (2007). Distinct vascular endothelial growth factor signals for lymphatic vessel enlargement and sprouting.. J Exp Med.

[20] Huggenberger R, Ullmann S, Proulx ST, Pytowski B, Alitalo K (2010). Stimulation of lymphangiogenesis via VEGFR-3 inhibits chronic skin inflammation.. J Exp Med.

[21] O'Farrell AM, Abrams TJ, Yuen HA, Ngai TJ, Louie SG (2003). SU11248 is a novel FLT3 tyrosine kinase inhibitor with potent activity in vitro and in vivo.. Blood.

[22] Pytowski B, Goldman J, Persaud K, Wu Y, Witte L (2005). Complete and specific inhibition of adult lymphatic regeneration by a novel VEGFR-3 neutralizing antibody.. J Natl Cancer Inst.

[23] Niessen K, Zhang G, Ridgway JB, Chen H, Yan M (2010). ALK1 signaling regulates early postnatal lymphatic vessel development.. Blood.

[24] Ledgerwood LG, Lal G, Zhang N, Garin A, Esses SJ (2008). The sphingosine 1-phosphate receptor 1 causes tissue retention by inhibiting the entry of peripheral tissue T lymphocytes into afferent lymphatics.. Nat Immunol.

[25] Ebos JM, Lee CR, Christensen JG, Mutsaers AJ, Kerbel RS (2007). Multiple circulating proangiogenic factors induced by sunitinib malate are tumor-independent and correlate with antitumor efficacy.. Proc Natl Acad Sci U S A.

[26] Tammela T, Zarkada G, Wallgard E, Murtomaki A, Suchting S (2008). Blocking VEGFR-3 suppresses angiogenic sprouting and vascular network formation.. Nature.

[27] Zumsteg A, Baeriswyl V, Imaizumi N, Schwendener R, Ruegg C (2009). Myeloid cells contribute to tumor lymphangiogenesis.. PLoS One.

[28] Paik SG, Fleischer N, Shin SI (1980). Insulin-dependent diabetes mellitus induced by subdiabetogenic doses of streptozotocin: obligatory role of cell-mediated autoimmune processes.. Proc Natl Acad Sci U S A.

[29] Maki T, Gottschalk R, Ogawa N, Monaco AP (2005). Prevention and cure of autoimmune diabetes in nonobese diabetic mice by continuous administration of FTY720.. Transplantation.

[30] Fu F, Hu S, Deleo J, Li S, Hopf C (2002). Long-term islet graft survival in streptozotocin- and autoimmune-induced diabetes models by immunosuppressive and potential insulinotropic agent FTY720.. Transplantation.

[31] Liao S, Ruddle NH (2006). Synchrony of high endothelial venules and lymphatic vessels revealed by immunization.. J Immunol.

[32] Allen SJ, Crown SE, Handel TM (2007). Chemokine: receptor structure, interactions, and antagonism.. Annu Rev Immunol.

[33] Martin AP, Rankin S, Pitchford S, Charo IF, Furtado GC (2008). Increased expression of CCL2 in insulin-producing cells of transgenic mice promotes mobilization of myeloid cells from the bone marrow, marked insulitis, and diabetes.. Diabetes.

[34] Jansen A, Homo-Delarche F, Hooijkaas H, Leenen PJ, Dardenne M (1994). Immunohistochemical characterization of monocytes-macrophages and dendritic cells involved in the initiation of the insulitis and beta-cell destruction in NOD mice.. Diabetes.

[35] Jun HS, Santamaria P, Lim HW, Zhang ML, Yoon JW (1999). Absolute requirement of macrophages for the development and activation of beta-cell cytotoxic CD8+ T-cells in T-cell receptor transgenic NOD mice.. Diabetes.

[36] Baluk P, Tammela T, Ator E, Lyubynska N, Achen MG (2005). Pathogenesis of persistent lymphatic vessel hyperplasia in chronic airway inflammation.. J Clin Invest.

[37] Maruyama K, Ii M, Cursiefen C, Jackson DG, Keino H (2005). Inflammation-induced lymphangiogenesis in the cornea arises from CD11b-positive macrophages.. J Clin Invest.

[38] Dietrich T, Bock F, Yuen D, Hos D, Bachmann BO (2010). Cutting edge: lymphatic vessels, not blood vessels, primarily mediate immune rejections after transplantation.. J Immunol.

[39] Kataru RP, Jung K, Jang C, Yang H, Schwendener RA (2009). Critical role of CD11b+ macrophages and VEGF in inflammatory lymphangiogenesis, antigen clearance, and inflammation resolution.. Blood.

[40] Schoppmann SF, Birner P, Stockl J, Kalt R, Ullrich R (2002). Tumor-associated macrophages express lymphatic endothelial growth factors and are related to peritumoral lymphangiogenesis.. Am J Pathol.

[41] Guo R, Zhou Q, Proulx ST, Wood R, Ji RC (2009). Inhibition of lymphangiogenesis and lymphatic drainage via vascular endothelial growth factor receptor 3 blockade increases the severity of inflammation in a mouse model of chronic inflammatory arthritis.. Arthritis Rheum.

[42] Huggenberger R, Siddiqui SS, Brander D, Ullmann S, Zimmermann K (2011). An important role of lymphatic vessel activation in limiting acute inflammation.. Blood.

[43] Yin N, Zhang N, Xu J, Shi Q, Ding Y (2011). Targeting lymphangiogenesis after islet transplantation prolongs islet allograft survival.. Transplantation.

[44] Kerjaschki D, Regele HM, Moosberger I, Nagy-Bojarski K, Watschinger B (2004). Lymphatic neoangiogenesis in human kidney transplants is associated with immunologically active lymphocytic infiltrates.. J Am Soc Nephrol.

[45] Nykanen AI, Sandelin H, Krebs R, Keranen MA, Tuuminen R (2010). Targeting lymphatic vessel activation and CCL21 production by vascular endothelial growth factor receptor-3 inhibition has novel immunomodulatory and antiarteriosclerotic effects in cardiac allografts.. Circulation.

[46] Kang S, Lee SP, Kim KE, Kim HZ, Memet S (2009). Toll-like receptor 4 in lymphatic endothelial cells contributes to LPS-induced lymphangiogenesis by chemotactic recruitment of macrophages.. Blood.

[47] Vigl B, Aebischer D, Nitschke M, Iolyeva M, Rothlin T (2011). Tissue inflammation modulates gene expression of lymphatic endothelial cells and dendritic cell migration in a stimulus-dependent manner.. Blood.

[48] Kerjaschki D (2005). The crucial role of macrophages in lymphangiogenesis.. J Clin Invest.

[49] Kubota Y, Takubo K, Shimizu T, Ohno H, Kishi K (2009). M-CSF inhibition selectively targets pathological angiogenesis and lymphangiogenesis.. J Exp Med.

[50] Olsson AK, Dimberg A, Kreuger J, Claesson-Welsh L (2006). VEGF receptor signalling - in control of vascular function.. Nat Rev Mol Cell Biol.

[51] Edirisinghe I, Yang SR, Yao H, Rajendrasozhan S, Caito S (2008). VEGFR-2 inhibition augments cigarette smoke-induced oxidative stress and inflammatory responses leading to endothelial dysfunction.. FASEB J.

[52] Hamrah P, Chen L, Cursiefen C, Zhang Q, Joyce NC (2004). Expression of vascular endothelial growth factor receptor-3 (VEGFR-3) on monocytic bone marrow-derived cells in the conjunctiva.. Exp Eye Res.

[53] Leclers D, Durand K, Cook-Moreau J, Rabinovitch-Chable H, Sturtz FG (2006). VEGFR-3, VEGF-C and VEGF-D mRNA quantification by RT-PCR in different human cell types.. Anticancer Res.

[54] Faivre S, Demetri G, Sargent W, Raymond E (2007). Molecular basis for sunitinib efficacy and future clinical development.. Nat Rev Drug Discov.

[55] Hipp MM, Hilf N, Walter S, Werth D, Brauer KM (2008). Sorafenib, but not sunitinib, affects function of dendritic cells and induction of primary immune responses.. Blood.

[56] Louvet C, Szot GL, Lang J, Lee MR, Martinier N (2008). Tyrosine kinase inhibitors reverse type 1 diabetes in nonobese diabetic mice.. Proc Natl Acad Sci U S A.

[57] Mandala S, Hajdu R, Bergstrom J, Quackenbush E, Xie J (2002). Alteration of lymphocyte trafficking by sphingosine-1-phosphate receptor agonists.. Science.

[58] Yoon CM, Hong BS, Moon HG, Lim S, Suh PG (2008). Sphingosine-1-phosphate promotes lymphangiogenesis by stimulating S1P1/Gi/PLC/Ca2+ signaling pathways.. Blood.

